# Flow-induced voltage generation over monolayer graphene in the presence of herringbone grooves

**DOI:** 10.1186/1556-276X-8-487

**Published:** 2013-11-20

**Authors:** Seung Ho Lee, Young Bok (Abraham) Kang, Wonsuk Jung, Yousung Jung, Soohyun Kim, Hongseok (Moses) Noh

**Affiliations:** 1 Department of Mechanical Engineering, Korea Advanced Institute of Science and Technology, Daejeon 305-701, Republic of Korea; 2 Graduate School of Energy, Environment, Water, and Sustainability, Korea Advanced Institute of Science and Technology, Daejeon 305-701, Republic of Korea; 3 Mechanical Engineering and Mechanics, Drexel University, Philadelphia, PA 19104, USA

**Keywords:** Graphene, Voltage generation, Herringbone grooves, Transverse flow, Microchannel

## Abstract

While flow-induced voltage over a graphene layer has been reported, its origin remains unclear. In our previous study, we suggested different mechanisms for different experimental configurations: phonon dragging effect for the parallel alignment and an enhanced out-of-plane phonon mode for the perpendicular alignment (Appl. Phys. Lett. 102:063116, 2011). In order to further examine the origin of flow-induced voltage, we introduced a transverse flow component by integrating staggered herringbone grooves in the microchannel. We found that the flow-induced voltage decreased significantly in the presence of herringbone grooves in both parallel and perpendicular alignments. These results support our previous interpretation.

## Background

Over the past decade, theoretical and experimental studies have demonstrated that a voltage is generated when carbon nanotubes (CNT) and graphene surfaces are exposed to fluid flows [1-8]. Kral and Shapiro first proposed theoretical mechanisms for flow-induced current generation within metallic single-walled carbon nanotubes (m-SWCNTs) [9]. This flow-induced voltage was then experimentally demonstrated for the first time by Sood et al., who used a SWCNT film deposited between electrodes immersed in a flowing liquid [1]. Similar experiments were conducted with multiwalled carbon nanotubes (MWCNTs) [3]. The aligned MWCNTs were found to generate voltages 15 times higher than SWCNTs. We also reported that semiconducting single-walled carbon nanotubes (s-SWCNTs) can produce voltages three times higher than m-SWCNTs in flowing liquids [5]. Similar phenomena were observed on graphene surfaces on exposure to fluid flows. Dhiman et al. reported that a graphene surface could generate a peak voltage of approximately 25 mV in fluid flows [6]. They proposed surface ion hopping as the major mechanism for the flow-induced voltage generation.

However, the precise mechanism of flow-induced voltage generation over graphene and CNT surfaces remains unclear. To understand the origin of the flow-induced voltage, we previously conducted experiments with two different electrode-flow configurations: electrodes aligned parallel and perpendicular to the fluid flow. These experimental results suggested that the main mechanism for parallel alignment was the ‘phonon dragging model’ [9], while that for perpendicular alignment was the ‘enhanced out-of-plane phonon mode’ [8].

**Figure 1:**
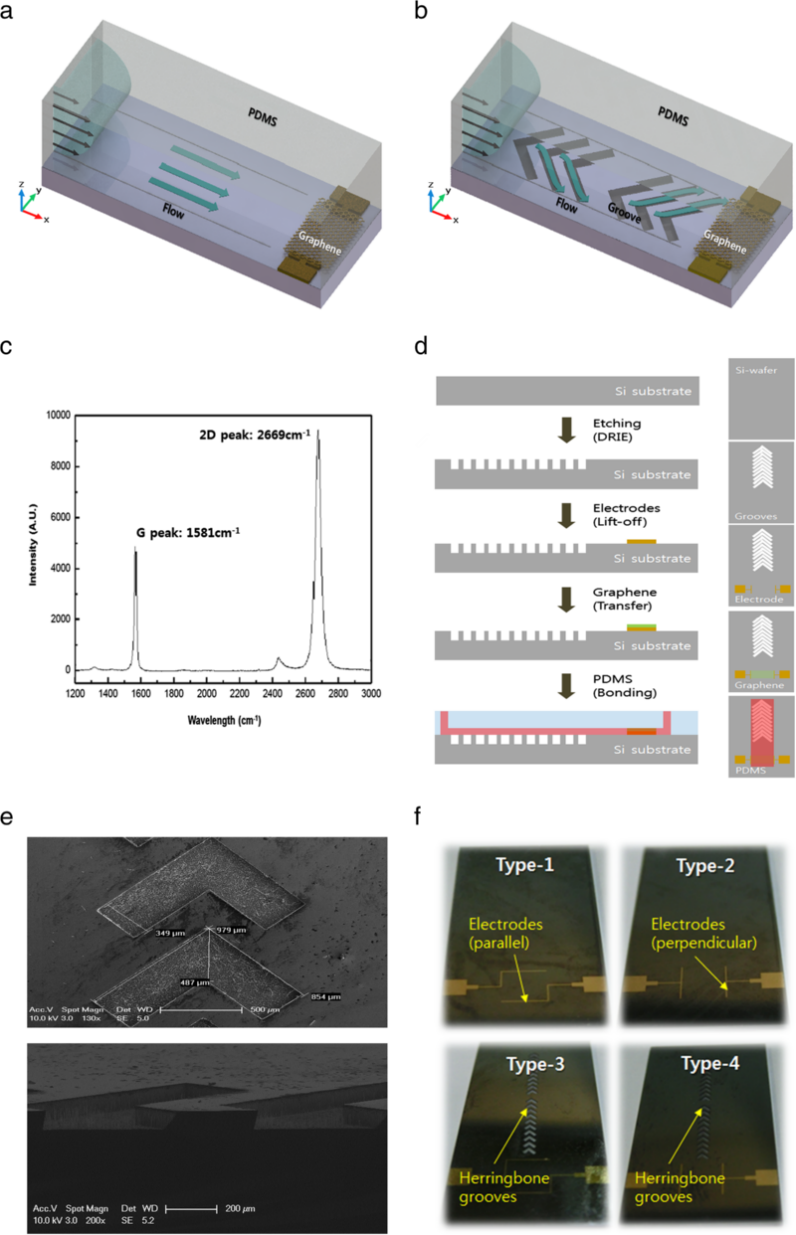
**Device preparation. (a**, **b)** Schematic illustration of the test device without and with herringbone grooves. **(c)** Raman spectra of monolayered graphene. **(d)** Fabrication and assembly. **(e)** SEM images of herringbone grooves. **(f)** Four different types of device configurations according to the electrode-flow alignment and the presence of herringbone grooves.

Here, we modified the flow to have a transverse component by introducing staggered herringbone grooves in the microchannel to further examine the origin of the induced voltage in Figure 1a,b. The staggered herringbone grooves enable rapid mixing in the microchannel by creating transverse flows [10,11]. Note that the *x*-direction indicates the longitudinal flow direction along the channel, while the *y*-direction indicates the transverse or lateral direction of the channel. Flow-induced voltages measured in devices with and without herringbone grooves were analyzed to examine the effects of the transverse flow component on voltage generation. The effects of flow rate and electrode-flow alignment were also investigated. The results suggested that flow-induced voltage generation with parallel and perpendicular alignments of the electrode with respect to the flow direction is due to different mechanisms, supporting our previous interpretation [8].

## Methods

A monolayer of graphene was grown separately on Cu foil in a chemical vapor deposition chamber, as reported previously [12,13]. It was verified that the graphene was a monolayer using Raman spectroscopy (the ratio of *G* and 2*D* peaks was 2 as shown in Figure 1c) [14].

The fabrication process for the device is shown in Figure 1d. To make the herringbone grooves in a silicon wafer, we used deep reactive ion etching (DRIE) [15,16]. There were 20 grooves with a width of 350 μm and a depth of 100 μm (see the scanning electron microscopy (SEM) image in Figure 1e). Each groove has staggered lengths of 865 μm and 1,000 μm. The grooves were designed to be at an angle of 45° to the channel wall and were spaced with an interval of 840 μm (center to center) along the length of the channel. The electrodes were then fabricated on the Si wafer with grooves using a lift-off technique [17]. A 10-nm-thick Cr layer and a 40-nm-thick Au layer were deposited sequentially on a predefined photoresist layer on the Si wafer to form the electrode patterns. After defining the electrodes, the wafer was diced into smaller substrates (15 mm × 20 mm). The graphene monolayer was then transferred onto the Si wafer and placed between the electrodes. The resistance of the graphene was about 1 kΩ.

Finally, the Si wafer with grooves, electrodes, and graphene was bonded to a polydimethylsiloxane (PDMS) layer, which had a fluidic channel of 100 μm in height, 1.5 mm in width, and 20 mm in length defined by replica molding. The PDMS layer was sealed to the Si surface by oxygen plasma treatment. Four types of samples were prepared in Figure 1f:

• *Type 1*: the electrodes aligned parallel to the flow in the absence of grooves

• *Type 2*: the electrodes aligned perpendicular to the flow in the absence of grooves

• *Type 3*: the electrodes aligned parallel to the flow in the presence of grooves

• *Type 4*: the electrodes aligned perpendicular to the flow in the presence of grooves

A syringe pump (Legato 180; KD Scientific, Holliston, MA, USA) was used to inject fluid through the PDMS microchannel. The flow-induced voltage over the graphene was measured using a digital multimeter (DM 2002; Keithley Instruments, Cleveland, OH, USA). All experiments were carried out at room temperature (25°C).

## Results and discussion

**Figure 2:**
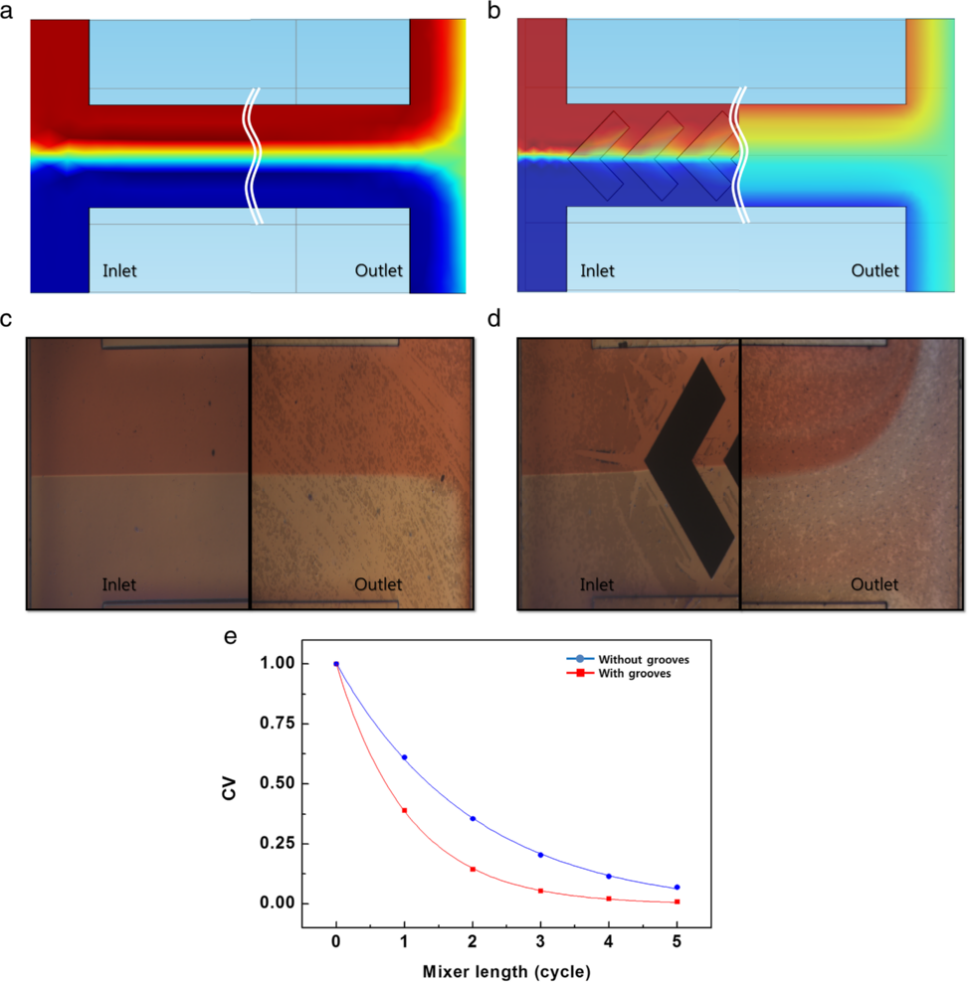
**Simulated and measured mixing performance. (a)** Simulated mixing performance in the absence of herringbone grooves. **(b)** Simulated mixing performance in the presence of herringbone grooves. **(c)** Actual mixing result in the absence of herringbone grooves. **(d)** Actual mixing result in the presence of herringbone grooves. **(e)** Coefficient of variation with and without herringbone grooves at a flow rate of 100 μL/min.

Prior to measuring flow-induced voltage, we investigated the mixing performance of the herringbone grooves. Figure 2a,b shows the simulation results of mixing between pure water and dyed water without and with herringbone grooves, respectively. A 3-D numerical simulation was performed using COMSOL Multiphysics (ver. 4.3a). The simulation geometry was identical to the actual microchannel device. Figure 2c,d shows the actual experimental data. Two streams of liquid (pure water and red dyed water) were injected into the microchannel via two inlets using a syringe pump. In the absence of herringbone grooves, only a minimal amount of mixing due to thermal diffusion was observed at the outlet of the channel in both simulated and experimental data. On the other hand, significantly more mixing was observed in the device with herringbone grooves. Mixing performance was also evaluated from the coefficient of variation (CV) [18], which is a normalized measure of dispersion of a probability distribution. The CV of concentration is considered a good measure of mixing quality. A positive value (approximately 1.0) indicates no mixing, and a value of 0 indicates complete mixing. As mixing progressed, the CV decayed exponentially from 1 to 0. We compared the mixing performance of the channels with and without grooves at various flow rates. At a flow rate of 100 μL/min, the channel with grooves (red line) showed better mixing performance (lower CV) than the channel without grooves (blue line in Figure 2e). The number of mixing cycles required for the transition from CV = 1 to CV = 0.1 was reduced from 4 to 2 cycles by the presence of grooves. These mixing results indicate that a transverse flow component was induced by the herringbone grooves.

**Figure 3:**
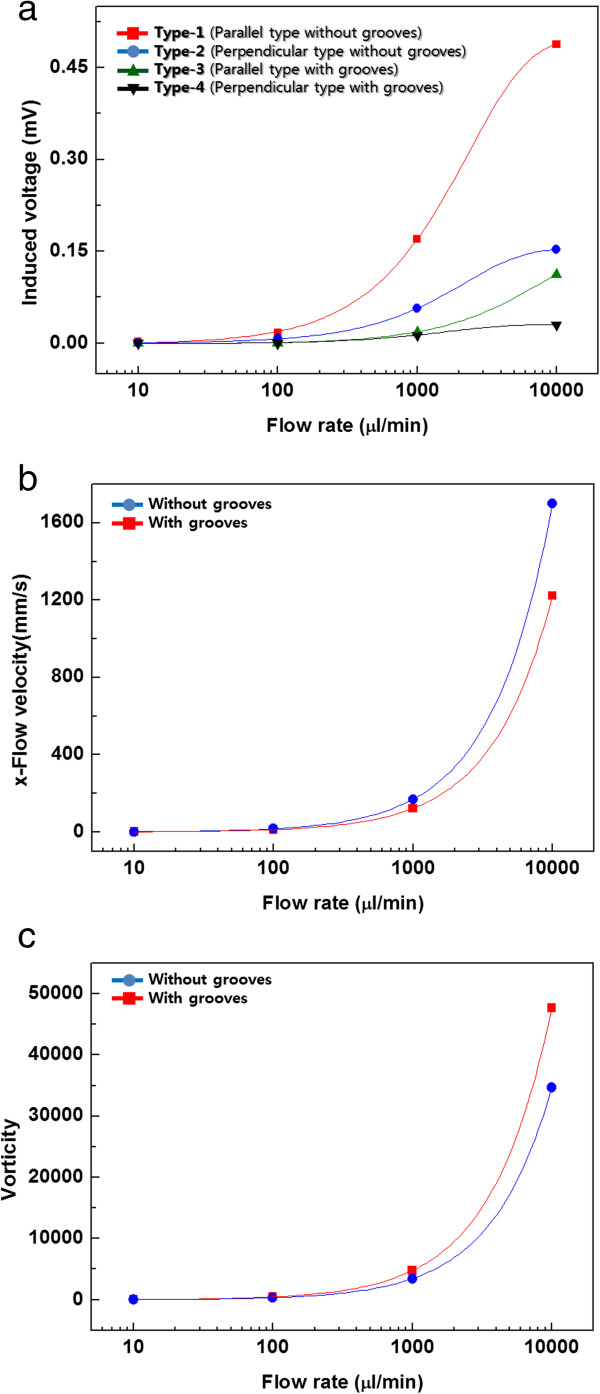
**Flow-induced voltage for four different types of devices. (a)** Flow-induced voltage with flow rate, **(b)***x*-directional flow velocity (longitudinal, flow direction), and **(c)** vorticity for devices with and without herringbone grooves.

Figure 3a shows the flow-induced voltage as a function of flow rate for the four different configurations tested in this study. Before discussing the effect of herringbone grooves, let us compare the two different electrode-flow alignments in the absence of herringbone grooves. Previous studies have indicated that a flow-induced voltage was generated only when the electrodes were aligned parallel to the flow (type 1), while no voltage was generated when the electrodes were aligned perpendicular to the flow (type 2) [1,6]. As shown in Figure 3a, however, a flow-induced voltage was generated with the electrodes aligned perpendicular to the flow (type 2). At a flow rate of 1,000 μL/min, the induced voltage (0.17 mV) with the parallel alignment (type 1) was three times higher than that (0.057 mV) of the perpendicular alignment (type 2). With an increase in the flow rate to 10,000 μL/min, the voltage also increased to 0.49 mV (type 1) and 0.15 mV (type 2). Previously, we suggested that different mechanisms are responsible for voltage generation in the case of parallel and perpendicular alignments [8]. When the electrodes are aligned parallel to the flow direction, charge carriers (electrons) localized on the graphene surface can be dragged along with the flow, producing flow velocity-dependent electricity. However, this mechanism does not explain voltage generation with perpendicular alignment. When the electrodes are aligned perpendicular to the flow direction, the momentum of the flowing liquid is transferred to the graphene and increases the amplitudes of spontaneous fluctuations in the graphene. This is what we called enhanced out-of-plane phonon mode, resulting in reorganization of the structure of interfacial water molecules, causing instantaneous potential differences even along the direction perpendicular to the flow [8]. Experimental data presented in Figure 3a confirm that flow-induced voltage generation is observed in the perpendicular alignment due to the enhanced out-of-plane phone mode. The flow-induced voltage generated in the parallel alignment is likely the outcome of combined effects of both phonon dragging and enhanced out-of-plane phonon modes, although the former seems to be more dominant than the latter.

Now, let us consider the effects of the herringbone grooves in both parallel and perpendicular alignments (type 3 and type 4 in Figure 3a). In the case of the parallel alignment, a significant decrease in the induced voltage was observed with the herringbone grooves. At a flow rate of 1,000 μL/min, the voltage decreased by almost tenfold, from 0.17 mV (type 1) to 0.018 mV (type 3). At a flow rate of 10,000 μL/min, the induced voltage dropped from 0.49 mV (type 1) to 0.11 mV (type 3). To understand why the presence of herringbone grooves significantly decreased the induced voltage, we performed simulation studies on flow velocity and vorticity. Figure 3b shows the flow velocity in the *x*-direction (longitudinal, flow direction) over the graphene surface as a function of flow rate. While the volumetric flow rate was kept constant for both type 1 and type 3, the flow velocity in the *x*-direction decreased when herringbone grooves were added. At a flow rate of 1,000 μL/min, the flow velocity in the *x*-direction decreased from 169.36 to 122.27 mm/s. This was due to the presence of transverse flow generated by the grooves in the microfluidic channel. The decrease in flow velocity (*x*-direction) resulted in a reduced electron dragging effect, and as a result, the flow-induced voltage decreased. Moreover, vorticity increased in the presence of groove as shown in Figure 3c. At a flow rate of 1,000 μL/min, the vorticity in the channel with herringbone grooves was 38% higher than that in the channel without grooves. Vorticity, the curl of the velocity vector, indicates local spinning or rotational motion of a fluid. It seems that the increased vorticity of fluid disturbed the directional electron dragging, resulting in a further decrease in voltage generation. Therefore, the significant decrease in the induced voltage in the presence of herringbone grooves is due to the combined effects of reduced flow velocity and increased vorticity.

In the case of perpendicular alignment, a significant decrease in the induced voltage was observed as well when herringbone grooves were included. At a flow rate of 1,000 μL/min, the voltage decreased by fourfold, from 0.057 mV (type 2) to 0.013 mV (type 4). At a flow rate of 10,000 μL/min, the induced voltage dropped from 0.15 mV (type 2) to 0.03 mV (type 4). At a glance, this result may be surprising because one may think that the increased transverse flow along the *y*-direction would induce a stronger phonon dragging effect. However, simulation data revealed that the flow velocity in the *y*-direction along the electron path between the electrodes was appreciably small compared to that in the *x*-direction. As a result, any added electron dragging effect due to the increase in transverse flow was buried in the effect of the overall flow momentum decrease due to the decrease in *x*-directional flow velocity in Figure 3b. Moreover, the increased vorticity seems to interfere with the out-of-plane phonon mode, minimizing the momentum transfer from the fluid flow in Figure 3c. In summary, the significant decrease in the induced voltage in the presence of herringbone grooves is because of the overall flow momentum decrease due to the decrease in *x*-directional flow and increased vorticity.

**Figure 4:**
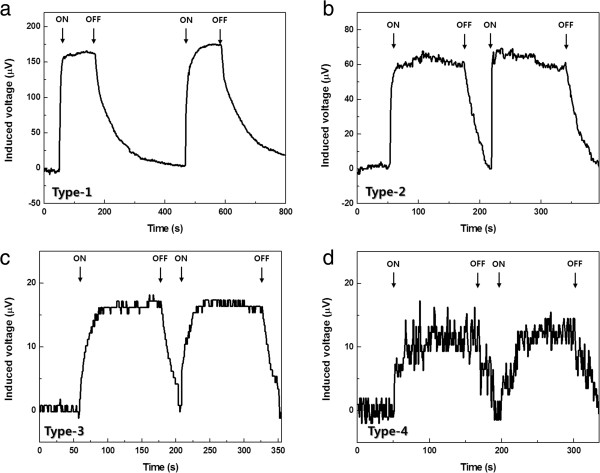
**Flow-induced voltage with time. (a)** Parallel alignment without herringbone grooves. **(b)** Perpendicular alignment without herringbone grooves. **(c)** Parallel alignment with herringbone grooves. **(d)** Perpendicular alignment with herringbone grooves.

Figure 4 shows the flow-induced voltage generation with time at a fixed flow rate (1,000 μL/min) for all four configurations. It is notable that the signals for the perpendicular alignment (in Figure 4b,d) have more noise/oscillation than those for the parallel alignment (in Figure 4a,c). This difference seemed to arise from the distinct voltage generation mechanisms. As the out-of-plane phonon mode is produced by momentum transfer from the flowing fluid to the graphene layer, the induced voltage tends to show greater oscillation than the signal obtained in phonon dragging mode. This signal oscillation is amplified with the herringbone grooves due to the increased vorticity in the fluid flow in Figure 4d. These data also support our previously proposed different mechanisms for flow-induced voltage generation according to the electrode-flow alignment.

## Conclusions

In conclusion, we investigated flow-induced voltage generation over a graphene monolayer in the presence of staggered herringbone grooves to better understand the origin of the voltage generated. The flow-induced voltage decreased significantly in the presence of herringbone grooves in both parallel and perpendicular alignments. The numerical simulation study revealed that the presence of herringbone grooves decreased longitudinal flow velocity while increasing transverse flow and vorticity. As a result, the directional charge dragging effect was significantly reduced in the parallel alignment, resulting in decreased voltage generation. In the case of the perpendicular alignment, the momentum transfer from the fluid flow to the graphene (out-of-plane phonon mode) was affected by the decreased flow velocity and increased vorticity, causing the voltage generation to drop. We also found that the voltage signal with the perpendicular alignment showed a bigger oscillation than that of the parallel type and that the signal oscillation was amplified by the herringbone groove. These data support that the mechanism for flow-induced voltage in perpendicular alignment is different from the parallel alignment case and that it is related to momentum transfer from the fluid flow. Taken together, the experimental data presented here support our previous proposal regarding the distinct flow-induced voltage generation mechanisms for parallel and perpendicular alignments.

## Competing interests

The authors declare that they have no competing interests.

## Authors’ contributions

SHL conducted and participated in the entire work from preparation of the devices to experimental characterization and numerical simulations. He prepared the current manuscript as the first author. YBK and WJ participated in the design, fabrication, and testing of the herringbone mixer device and also in the manuscript preparation. YJ participated in the discussion and interpretation of the experimental results for the flow-induced voltage generation. SK and HN supervised the entire work and participated in the manuscript preparation. All authors read and approved the final manuscript.
